# Robot-assisted minimally invasive thoraco-laparoscopic esophagectomy versus open transthoracic esophagectomy for resectable esophageal cancer, a randomized controlled trial (ROBOT trial)

**DOI:** 10.1186/1745-6215-13-230

**Published:** 2012-11-30

**Authors:** Pieter C van der Sluis, Jelle P Ruurda, Sylvia van der Horst, Roy JJ Verhage, Marc GH Besselink, Margriet JD Prins, Leonie Haverkamp, Carlo Schippers, Inne HM Borel Rinkes, Hans CA Joore, Fiebo JW ten Kate, Hendrik Koffijberg, Christiaan C Kroese, Maarten S van Leeuwen, Martijn PJK Lolkema, Onne Reerink, Marguerite EI Schipper, Elles Steenhagen, Frank P Vleggaar, Emile E Voest, Peter D Siersema, Richard van Hillegersberg

**Affiliations:** 1 Department of Surgery, G04.228, University Medical Center Utrecht, Heidelberglaan 100, Utrecht, CX, 3584, the Netherlands; 2 Department of Intensive Care Medicine, University Medical Center Utrecht, Heidelberglaan 100, Utrecht, CX, 3584, the Netherlands; 3 Department of Pathology, University Medical Center Utrecht, Heidelberglaan 100, Utrecht, CX, 3584, the Netherlands; 4 Julius Center for Health Sciences and Primary Care, University Medical Center Utrecht, Heidelberglaan 100, Utrecht, CX, 3584, the Netherlands; 5 Department of Anesthesiology, University Medical Center Utrecht, Heidelberglaan 100, Utrecht, CX, 3584, the Netherlands; 6 Department of Radiology, University Medical Center Utrecht, Heidelberglaan 100, Utrecht, CX, 3584, the Netherlands; 7 Department of Medical Oncology, University Medical Center Utrecht, Heidelberglaan 100, Utrecht, CX, 3584, the Netherlands; 8 Department of Radiotherapy, University Medical Center Utrecht, Heidelberglaan 100, Utrecht, CX, 3584, the Netherlands; 9 Department of Gastroenterology and Hepatology, University Medical Center Utrecht, Heidelberglaan 100, Utrecht, CX, 3584, the Netherlands

## Abstract

**Background:**

For esophageal cancer patients, radical esophagolymphadenectomy is the cornerstone of multimodality treatment with curative intent. Transthoracic esophagectomy is the preferred surgical approach worldwide allowing for *en-bloc* resection of the tumor with the surrounding lymph nodes. However, the percentage of cardiopulmonary complications associated with the transthoracic approach is high (50 to 70%).

Recent studies have shown that robot-assisted minimally invasive thoraco-laparoscopic esophagectomy (RATE) is at least equivalent to the open transthoracic approach for esophageal cancer in terms of short-term oncological outcomes. RATE was accompanied with reduced blood loss, shorter ICU stay and improved lymph node retrieval compared with open esophagectomy, and the pulmonary complication rate, hospital stay and perioperative mortality were comparable. The objective is to evaluate the efficacy, risks, quality of life and cost-effectiveness of RATE as an alternative to open transthoracic esophagectomy for treatment of esophageal cancer.

**Methods/design:**

This is an investigator-initiated and investigator-driven monocenter randomized controlled parallel-group, superiority trial. All adult patients (age ≥18 and ≤80 years) with histologically proven, surgically resectable (cT1-4a, N0-3, M0) esophageal carcinoma of the intrathoracic esophagus and with European Clinical Oncology Group performance status 0, 1 or 2 will be assessed for eligibility and included after obtaining informed consent. Patients (*n* = 112) with resectable esophageal cancer are randomized in the outpatient department to either RATE (*n* = 56) or open three-stage transthoracic esophageal resection (*n* = 56). The primary outcome of this study is the percentage of overall complications (grade 2 and higher) as stated by the modified Clavien–Dindo classification of surgical complications.

**Discussion:**

This is the first randomized controlled trial designed to compare RATE with open transthoracic esophagectomy as surgical treatment for resectable esophageal cancer. If our hypothesis is proven correct, RATE will result in a lower percentage of postoperative complications, lower blood loss, and shorter hospital stay, but with at least similar oncologic outcomes and better postoperative quality of life compared with open transthoracic esophagectomy. The study started in January 2012. Follow-up will be 5 years. Short-term results will be analyzed and published after discharge of the last randomized patient.

**Trial registration:**

Dutch trial register: NTR3291 ClinicalTrial.gov: NCT01544790

## Background

In 2008 an estimated 482,300 people were diagnosed with esophageal cancer, and 406,800 patients died of the disease worldwide [1]. Radical esophagolymphadenectomy is the cornerstone of the multimodality treatment with curative intent [2-5].

Transthoracic esophagectomy is the preferred surgical approach worldwide allowing for *en-bloc* resection of the tumor with the surrounding para-tracheal, subcarinal and para-esophageal lymph nodes [6,7]. However, the percentage of cardiopulmonary complications associated with the transthoracic approach is high (50 to 70%) [6].

Minimally invasive esophagectomy was designed to reduce surgical trauma, resulting in lower morbidity and mortality rates. With regard to minimally invasive esophagectomy, review of the literature shows a substantial decrease in blood loss, postoperative complications and days of hospital stay, with comparable oncologic results [8-12].

In 2003 the robot-assisted thoraco-laparoscopic approach was developed at the University Medical Center Utrecht (UMCU), the Netherlands [13]. Robot-assisted thoraco-laparoscopic esophagectomy facilitates complex minimally invasive procedures with an enlarged, three-dimensional field of view. The articulated instruments allow dissection with seven degrees of freedom [13,14].

Until now there have been no prospective randomized controlled trials comparing robot-assisted minimally invasive esophagectomy with conventional open transthoracic esophagectomy. We present the protocol of the first randomized controlled trial comparing these two surgical approaches.

### Aim of the study

This is a randomized controlled parallel-group, superiority trial of robot-assisted thoraco-laparoscopic esophagectomy versus open three-stage transthoracic esophagectomy in patients with resectable intrathoracic esophageal cancer.

## Methods

### Objectives

Patients with resectable esophageal cancer are randomized at the outpatient department to either robot-assisted thoraco-laparoscopic esophagectomy or open three-stage transthoracic esophageal resection. The objective is to evaluate the efficacy, risks and cost-effectiveness of robot-assisted thoraco-laparoscopic esophagectomy as an alternative to open transthoracic esophagectomy as treatment for esophageal cancer. We hypothesize that robot-assisted minimally invasive thoraco-laparoscopic esophagectomy leads to a lower postoperative complication rate, less blood loss and a shorter hospital stay, with similar oncologic outcomes and better postoperative quality of life, compared with the open transthoracic esophagectomy (current reference standard of care).

### Study design

This is an investigator-initiated and investigator-driven randomized controlled parallel-group, superiority trial comparing robot-assisted thoraco-laparoscopic esophagectomy with traditional open three-stage transthoracic esophageal resection.

This study is conducted in accordance with the principles of the Declaration of Helsinki [15] and Good Clinical Practice Guidelines [16]. The independent ethics committee of the UMCU has approved the study. Written informed consent will be obtained from all participating patients. Clinical trial monitoring will be conducted by an independent data monitor (Julius Clinical Research, Zeist, the Netherlands).

### Study population

All adult patients (age ≥18 and ≤80 years) with histologically proven, surgically resectable (cT1-4a, N0-3, M0) squamous cell carcinoma, adenocarcinoma or undifferentiated esophageal carcinoma of the intrathoracic esophagus will be assessed for eligibility. Patients should have a performance status 0, 1 or 2 according to the European Clinical Oncology Group.

The Patients’ inclusion and exclusion criteria are:

Inclusion criteria

•Histologically proven squamous cell carcinoma, adenocarcinoma or undifferentiated carcinoma of the intrathoracic esophagus (including Siewert I and II).

•Surgically resectable (T1-4a, N0-3, M0).

•Age ≥18 and ≤80 years.

•European Clinical Oncology Group performance status 0,1 or 2.

•Written informed consent.

Exclusion criteria

•Carcinoma of the cervical esophagus.

•Carcinoma of the gastro-esophageal junction with the main part of the tumor in the gastric cardia (Siewert type III).

•Prior thoracic surgery at the right hemithorax or thoracic trauma.

### Study protocol

Patients are informed about the trial by one of our surgeons (RvH or JPR) at the outpatient department. After receiving the information, all patients have 1 week to consider their consent. After 1 week, patients are contacted by the coordinating researcher (PCvdS) to make an appointment to obtain and register informed consent.

After obtaining informed consent, randomization is done by computer-generated random numbers. Concealment of allocation is maintained by using sealed opaque envelopes. There is no blinding for the patient, surgeon and coordinating researcher because this is difficult in daily practice. However, the independent data monitoring safety committee is blinded to the allocated intervention. Within 1 week, patients will be informed about the allocated treatment. This study is completed funded by the Department of Surgery, UMCU. Multiple esophageal cancer biopsies for pathological analysis will be obtained through esophagogastroscopy, of which four biopsies will be snap frozen and stored for translational research. The physical status of the patient is assessed and preoperative testing is guided by institutional guidelines [17].

Neoadjuvant (radio)chemotherapy will be administered according to the current policy in the Netherlands and the UMCU [17]. Two additional blood samples will be obtained for translational research (proteomics) at the following times: before start of neoadjuvant treatment, the day of operation, after adjuvant treatment and with suspicion of recurrent disease.

After finishing preoperative neoadjuvant treatment, patients will be evaluated with a second computed tomography scan for metastases and resectability. When the tumor is considered to be resectable, patients will undergo the randomized intervention – either robot-assisted thoraco-laparoscopic esophagectomy or open three-stage transthoracic esophagectomy depending on randomization.

All resection specimens will be preserved and stored (biobank, tissue-microarray) for translational research.

The study started on 1 January 2012. Inclusion will take approximately 3 years. Follow-up for each patient will be 5 years. The total duration of the study will be 8 years.

### Surgery

All procedures (robot-assisted thoraco-laparoscopic esophagectomy or open transthoracic esophagectomy) will be carried out by the same experienced surgeons in the UMCU (JPR and RvH). All patients will receive an epidural catheter to provide adequate postoperative analgesia. Patients will be intubated with a left-sided double-lumen tube to enable selective desufflation of the right lung during the thoracic phase in both procedures.

Prophylactic antibiotics cefazolin (2,000 mg) and metronidazole (500 mg) will be administered 30 minutes prior to incision [14]. An intravenous injection of 10 mg/kg methylprednisolone will be administered 30 minutes prior to incision to minimize postoperative pulmonary complications [18]. During single-lung ventilation, a pressure-controlled ventilation strategy will be used with a maximum pressure of 20 cm H_2_O [19].

### Open three-stage transthoracic esophagectomy

The patient is placed in a left lateral decubitus position and the procedure commences with a right posterolateral thoracotomy. After incision and desufflation of the right lung, the pulmonary ligament is incised followed by identification of the azygos vein. The azygos vein is clipped and ligated at the level of the azygos arch. The thoracic duct is identified, clipped and ligated. The esophagus is resected *en bloc* with the surrounding mediastinal lymph nodes. The resected specimen will contain right-sided paratracheal (lymph node station 2R), tracheobronchial (lymph node station 4), aortopulmonary window (station 5), carinal (station 7) and peri-esophageal (station 8) lymph nodes [20].

Chest tubes are placed and the thoracotomy wound is closed using intracutaneous closure with absorbable sutures.

The patient is turned to a supine position for the abdominal phase via supra-umbilical laparotomy. The stomach is mobilized with special care for the gastroepiploic and short gastric vessels. The left gastric artery is identified, clipped and ligated. Lymph node dissection is performed around the celiac trunk and the lesser omentum. A linear stapler (GIATM 80, 3.8 mm; Covidien, Mansfield, MA, USA) is used to create a gastric conduit 4 cm wide, which is routinely oversewn [21]. The gastric conduit is pulled up through the mediastinum along the original anatomic tract of the esophagus with the aid of a plastic tube (laparoscopic camera bag). A cervical handsewn end-to-side anastomosis is created between the gastric tube and the cervical esophagus using a 3/0 polydioxanone single-layer running suture. A feeding jejunostomy is placed in the second loop after the ligament of Treitz for postoperative feeding. The abdomen is closed in layers with PDS loop for the fascia and skin intracutaneously with monocryl. Patients are transferred to the ICU after the surgical procedure.

### Robot-assisted minimally invasive thoraco-laparoscopic esophagectomy

**Figure 1:**
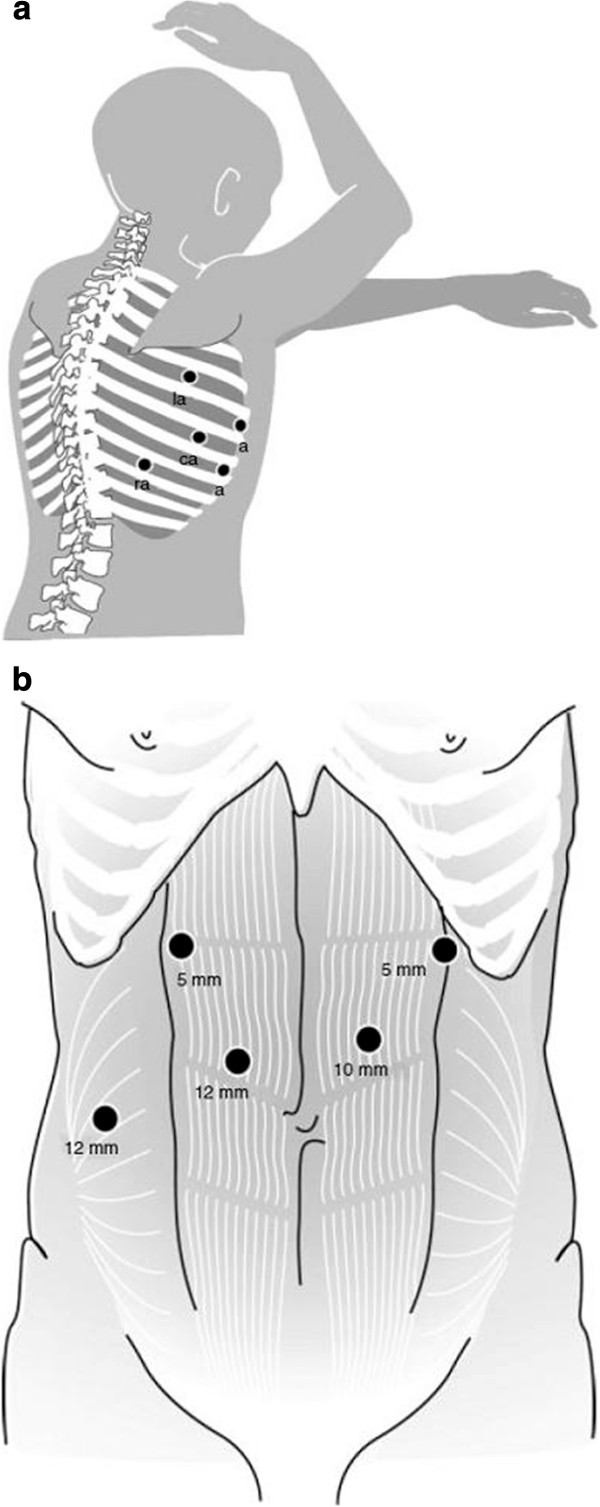
**Trocar arrangement during the robotassisted thoracoscopic phase.** (**a**) Trocar arrangement during robot-assisted thoracoscopic phase. La, left robotic arm (fourth intercostal space); a, assistant thoracoscopic working port (fifth and seventh intercostal space); ca, robotic camera arm (sixth intercostal space); ra, right robotic arm (eighth intercostal space) [14]. (**b**) Trocar arrangement during the laparoscopic abdominal phase. The camera was inserted through the 10 mm para-umbilical trocar port and two 5 mm trocars were used as laparoscopic working ports. The liver retractor was inserted through the 12 mm right para-rectal trocar port. The harmonic scalpel was inserted through the 12 mm paraumbilical port [14] .

Robot-assisted minimally invasive thoraco-laparoscopic esophagectomy was described previously [14]. For the thoracic phase, the patient is positioned in the left lateral decubitus position, tilted 45° towards the prone position. The robotic system (daVinci Si; Intuitive Surgical Inc., Sunnyvale, CA, USA) is brought into the field at the dorsocranial side of the patient. Three ports are placed for the robotic system as well as two thoracoscopic ports for the assisting surgeon (Figure 1a). After incision and installation of the operation robot and selective desufflation of the right lung, the pulmonary ligament is divided. Hereafter, the parietal pleura is dissected at the anterior side of the esophagus from the diaphragm up to the azygos arch. The azygos vein is ligated with Hem-o-lok (Teleflex Medical, Weck Drive, NC, USA) and divided [22]. Dissection of the parietal pleura is continued above the azygos arch to establish dissection of the right paratracheal lymph nodes. At the posterior side of the esophagus, the parietal pleura is dissected cranially to caudally along the azygos vein, including the thoracic duct. The thoracic duct is clipped with a 10 mm endoscopic clipping device (Endoclip™ II; Covidien) to prevent chylous leakage. To facilitate esophageal mobilization a penrose drain is placed around the esophagus to manipulate the esophagus for further mobilization. The esophagus is resected *en bloc* with the surrounding mediastinal lymph nodes. The resection specimen will contain the same lymph nodes as described for the open procedure.

For the abdominal phase, the patient is placed in a supine position. Figure 1b shows the position of the laparoscopic trocars. The lesser omentum is opened and transected closely to the liver, until the left crus of the diaphragm is reached. Hereafter, the greater gastric curvature is dissected using a harmonic ace. An abdominal lymphadenectomy is performed including lymph nodes surrounding the celiac trunk, along the left gastric and splenic artery and the lesser omental lymph nodes. The left gastric artery and vein are ligated with Hem-o-lok (Teleflex Medical) and transected at their origin.

Through a left-sided vertical incision along the sternocleidomastoid muscle, the cervical phase of esophagectomy is initiated to facilitate mobilization of the cervical esophagus. No formal cervical lymph node dissection is carried out, but macroscopically suspected cervical lymph nodes are dissected. The cervical esophagus is transected and a cord is attached to the specimen. The dissected esophagus *en bloc* with the surrounding lymph nodes are pulled down through the mediastinum under laparoscopic view.

Hereafter, the left para-umbilical trocar port is widened to a 5 to 7 cm transverse transabdominal incision. The resection specimen is removed through this incision with a wound drape (3M) to create the gastric conduit extracorporally. A linear stapler (GIATM 80, 3.8 mm; Covidien) is used to create a gastric conduit 4 cm wide, which is routinely oversewn [21]. The gastric conduit is pulled up through the mediastinum along the original anatomic tract of the esophagus with the aid of a plastic tube (laparoscopic camera bag). A cervical handsewn end-to-side anastomosis is created between the gastric tube and the cervical esophagus using a 3/0 polydioxanone single-layer running suture. A feeding jejunostomy is placed in the second loop after the ligament of Treitz for postoperative feeding. The abdomen is closed in layers with PDS loop for the fascia and skin intracutaneously with monocryl. Patients are transferred to the ICU after the surgical procedure.

### Outcome measurements

In terms of short-term oncological outcomes, we expect the robot-assisted esophagectomy to be equivalent to the open approach for survival but accompanied by fewer complications [9-12]. The primary outcome of this study is therefore the percentage of overall complications (grade 2 and higher) as stated by the modified Clavien–Dindo classification (MCDC) of surgical complications [23].

Secondary biochemical outcomes include individual components of the primary endpoint (major complications; MCDC grades II to IV), including myocardial infarction, anastomotic leakage (clinical or radiologic diagnosis), anastomotic stenosis, chylothorax (chylous leakage, presence of chylous in chest tubes or indication to start low fat (2%)-containing tube feeding; Vivonex® T.E.N.; Nestlé, Lutry, Switzerland ), gastric tube necrosis (proven by gastroscopy), pulmonary embolus, deep vein thrombosis, vocal cord palsy or paralysis. Minor complications (MCDC grade I) will also be recorded. These include, for example, wound infections, pleural effusions and delayed gastric emptying.

Length of ICU–medium care unit stay (days), length of hospital stay (days), in hospital mortality and mortality within 30 and 60 days will be reported. For all patients, the cause of death will be noted. If applicable, the results of the autopsy report will be noted. Two-year, 3-year and 5-year disease-free survival and overall survival will be reported.

The operation time is defined as time from incision until closure (minutes) for both the thoracic phase and the abdominal phase of the procedure. For the robotic procedure, the set-up time will be recorded separately. Unexpected events and complications occurring during the operation will be recorded (for example, hemorrhage requiring transfusion, perforation of other organs) as well as blood loss during operation (milliliters per phase). In the case of conversion to thoracotomy or laparotomy, the reason for conversion has to be explained (absolute numbers/percentage).

The resected specimen will be marked by the surgical team for the position of lymph node dissection. Evaluation will be performed by an experienced pathologist using standard protocols. Stage grouping will take place according to the Union Internationale Contre le Cancer protocol using the Tumor, Node, Metastasis-7 classification [24]. Exact localization of the lymph nodes is an essential part of the pathologic examination [20]. The pathology report contains the following parameters: site of tumor, type and gradation, extension in the esophageal wall, margins of the resection, extent of resection (R0 (oncological radical resection), R1 or R2) [25], lymph node status with the number of lymph nodes, tumor regression grade (according to Mandard and colleagues) [26], vaso-invasion and perineural growth. Quality control of pathology will be provided by a specialized gastrointestinal pathologist (FJWtK).

The type and dose of used analgesics will be noted during the hospital admission period. A visual analogue scale for pain will be noted at the following times: preoperatively, the first 10 days after surgery and a fixed period during follow-up (6 weeks, 6 months and yearly postoperatively up to 5 years).

The quality-of-life questionnaires Short Form-36, EORTC Quality-of-life Questionnaire Core 30 (Dutch), EORTC OES18 (Dutch) and EQ-5D (Appendices 1 and 2) will be required at the following times: preoperatively <5 days, and 6 weeks, 6 months and yearly up to 5 years postoperatively.

The approach for the cost analysis is comparing actual direct medical costs incurred with both strategies up until 5 years after the operation. Costs estimates will be based on the recorded volumes and unit costs associated with both procedures. This includes the costs of operation rooms, costs of hospital and ICU stays, and costs associated with complications and reoperations.

### Sample size calculation

**Figure 2:**
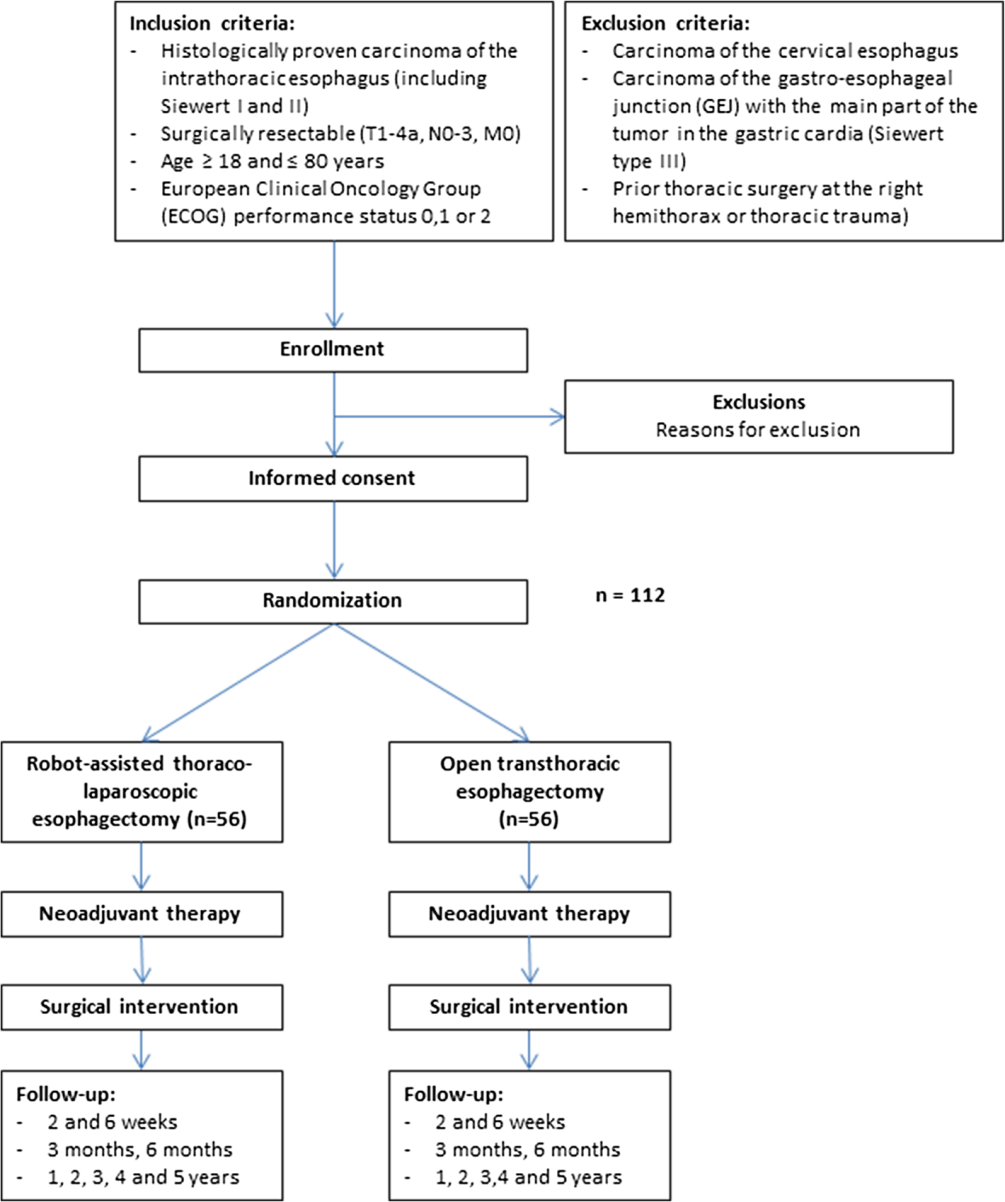
Flowchart for the ROBOT trial.

Hypothesis: Compared with an open transthoracic esophagectomy, robot-assisted thoraco-laparoscopic esophagectomy will result in a lower percentage of overall complications (MCDC grade 2 and higher). In a prospective analysis of our own series, MCDC grade 2 to 5 complications were observed in 69% of all patients who underwent robot-assisted thoraco-laparoscopic esophagectomy and in 91% of all patients who underwent open transthoracic esophagectomy in our won series in the UMCU (2003 to 2010). We calculated that 102 patients (51 in each arm) with resectable esophageal cancer would be required to detect this 22% reduction in the absolute risk of overall complications (from 91% to 69% of patients) based on a two-sided significance level (alpha) of 0.05 and a power of 0.80. An estimated compensation of 10% for drop out is included in the total number of patients, resulting in a total of 112 patients, 56 in each arm. Figure 2 visualizes the final design.

### Statistical analysis

All prospective data will be statistically analyzed using SPSS statistical software (SPSS Inc. Chicago, Illinois, USA. Data analysis will be performed in accordance with the intention-to-treat principle; additional per-protocol analysis will also be performed for tumor type, tumor stage and type of neoadjuvant treatment.

To evaluate significance of differences between the two groups, chi-squared and Fisher's exact tests will be used as appropriate for categorical variables, and the nonparametric Mann–Whitney U test for continuous variables.

To evaluate differences in disease-free and overall survival, Kaplan–Meier survival curves will be computed. Survival curves will be compared by log-rank test and multivariable analysis will be accomplished by the Cox regression model. The significance level will be set at 5%.

The approach for the cost analysis is comparing actual direct medical costs incurred with both strategies up until 5 years after the operation. Cost estimates will be based on the recorded volumes and unit costs associated with both procedures. This includes the costs of operation rooms, costs of hospital and ICU stays, and costs associated with complications and reoperations.

Pain scores will be analyzed using a linear mixed model using repeated-measures analysis. The quality-of-life questionnaires will be compared using covariance analysis (preoperative scores as covariables). The cost-effectiveness analysis will compare the mean costs and effects for both strategies and result in an incremental cost-effectiveness ratio. Uncertainty in the balance between costs and effects will be assessed with bootstrapping. A time horizon of 5 years will be applied, and costs and effects will be discounted according to Dutch guidelines.

If the baseline characteristics differ after randomization – that is, there is a lack of balance in the confounding factors – this will be corrected using multivariate analysis or using a net-benefit regression approach.

### Interim analysis

There will be one interim analysis. The stopping rule used for efficacy (that is, better outcome for the minimally invasive method as the primary endpoint) is the Peto approach, meaning *P* <0.001. The trial will not be stopped for futility (that is, no difference) as the robot-assisted minimally-invasive approach is being used by a growing numbers of centers worldwide and the outcome of all endpoints of this first randomized trial on this subject are relevant to healthcare professionals involved with this procedure in those hospitals. As is advised by the Dutch Central Committee on Research involving Human Subjects (CCMO), there is no formal stopping rule for harm.

After every 25 patients, individualized patient description charts including safety parameters will be presented to the Data Safety Monitoring Committee (DSMC). The DSMC will discuss these in a plenary or telephone conference with the study coordinator and principal investigator present. If the suspects are harmed (that is, worse outcome for the minimally invasive method) the DSMC will inform the trial research group. The trial research group will discuss in a plenary session together with the DSMC the potential harm per patient and will determine whether a relationship can be drawn between the minimally-invasive procedure and the adverse events. Consensus will be reached and the Medisch Ethische Toetsingscommissie will be informed.

## Discussion

To the best of our knowledge this is the first randomized controlled trial designed to compare robot-assisted minimally invasive thoraco-laparoscopic esophagectomy with open transthoracic esophagectomy as surgical treatment for resectable esophageal cancer.

In the 2010 revised Dutch esophageal carcinoma guidelines, open transthoracic esophagectomy is considered the first-choice procedure for patients with resectable esophageal carcinoma [17]. However, open transthoracic esophagectomy is accompanied with significant morbidity, predominantly through cardiopulmonary complications [6].

To reduce surgical trauma and morbidity of the open transthoracic esophagectomy, minimally invasive procedures have been designed to overcome this problem. However, conventional (thoraco)scopic surgery has some important limitations, such as a two-dimensional view, disturbed eye–hand coordination and limited degrees of freedom, which might limit the surgeon in performing an optimal radical esophageal and mediastinal lymph node dissection [27].

To overcome the limitations of conventional (thoraco)scopic surgery, the robot-assisted minimally invasive thoraco-laparoscopic esophagectomy was developed in the UMCU in 2003 [14]. Despite these unchallengeable technical advantages, evidence behind its superiority over the conventional open transthoracic esophagectomy is still lacking. From a systematic review, which included nine articles (130 cases) related to robot-assisted esophagectomy, it was concluded that robot-assisted esophagectomy was a feasible and safe technique [28]. In terms of short-term oncological outcomes, robot-assisted minimally invasive thoraco-laparoscopic esophagectomy was at least equivalent to the open transthoracic approach for esophageal cancer [9-12,28]. Robot-assisted minimally invasive thoraco-laparoscopic esophagectomy was accompanied with reduced blood loss, shorter ICU stay and improved lymph node retrieval compared with open esophagectomy, and the pulmonary complication rate, hospital stay and perioperative mortality were comparable [28]. Disadvantages of the robot-assisted thoraco-laparoscopic esophagectomy were reported to be a prolonged operative time and high costs consisting of acquisition of an operation robot and disposable tools [28].

The level of evidence for robot-assisted minimally invasive thoraco-laparoscopic esophagectomy is suboptimal and based on case series or expert opinions only (level 4 or 5) [28]. The systematic review strongly emphasized the need for well-conducted randomized controlled trials and long-term survival studies within a framework of measured and comparable outcomes to prove the superiority of robot-assisted minimally invasive thoraco-laparoscopic esophagectomy over the worldwide current standard open transthoracic esophagectomy [28].

Two articles about conventional minimally invasive esophagectomy were published recently [29,30]. Results from both articles show that minimally invasive esophagectomy in general is superior over open esophagectomy [29,30]. These results suggest that robot-assisted esophagectomy might also be superior. One could argue that the real question is whether robotic-assisted esophagectomy can improve outcomes when compared with coventional minimally invasive esophagectomy. However, with limited evidence available for the superiority of robot-assisted esophagectomy over open esophagectomy, it is too early to compare robot-assisted esophagectomy with conventional minimally invasive esophagectomy. Differences between these groups will probably be small and therefore large numbers of patients are needed to ensure enough statistical power. Such a clinical trial can only be performed worldwide in a multicenter fashion by surgeons who are experienced in both techniques to avoid bias.

The UMCU has the largest experience worldwide with robot-assisted thoraco-laparoscopic esophagectomy. Combined with a completed learning curve, our centre is considered the best place to compare robot-assisted esophagectomy with open transthoracic esophagectomy. We started this monocenter randomized controlled trial in 2012. This monocenter randomized controlled superiority trial can provide further evidence supporting the robot-assisted minimally invasive thoraco-laparoscopic esophagectomy as treatment for resectable esophageal cancer.

We anticipate that the inclusion for this study will take 3 years to complete. The study started in January 2012, and follow-up will be 5 years. Short-term results will be analyzed and published after discharge of the last randomized patient.

## Conclusion

This is the first randomized controlled trial designed to compare robot-assisted minimally invasive thoraco-laparoscopic esophagectomy with open transthoracic esophagectomy as surgical treatment for resectable esophageal cancer.

If our hypothesis is proven correct, robot-assisted minimally invasive thoraco-laparoscopic esophagectomy will result in a lower percentage of postoperative complications, lower blood loss and shorter hospital stay, but with at least similar oncologic outcomes and better postoperative quality of life compared with the open transthoracic esophagectomy (current standard).

## Trial status

Recruitment of patients started in January 2012.

## Abbreviations

DSMC: Data Safety Monitoring Committee; EORTC: European Organisation for Research and Treatment of Cancer; MCDC: Modified Clavien–Dindo classification; RATE: Robot-assisted minimally invasive thoraco-laparoscopic esophagectomy; UMCU: University Medical Center Utrecht.

## Competing interests

The authors declare that they have no competing interests.

## Authors’ contributions

PCvdS, JPR, SvdH, MJDP, LH, CS, RJJV, MGHB, and RvH were involved in developing the original study design and developed the research protocols. JPR, IHMBR, HCAJ, FJWtK, CCK, MSvL, MPJKL, OR, MEIS, ES, FPV, EEV, PDS and RvH are responsible for the clinical input. EK will be responsible for the economic and quality-of-life analysis. PCvdS, JPR and RvH drafted the paper. All authors provided input into revisions of the paper and have read and approved the final manuscript.
